# Single-cell transcriptome profile during the antibody decline phase following inactivated COVID-19 vaccination

**DOI:** 10.3389/fcimb.2025.1715387

**Published:** 2025-12-02

**Authors:** Zhongyi Zhu, Yaling Huang, Jiatong Sun, Meirong Li, Yong Chen, Fubaoqian Huang, Chuanyu Liu, Weijun Chen, Jinmin Ma

**Affiliations:** 1 College of Life Sciences, University of Chinese Academy of Sciences, Beijing, China; 2 Beijing Genomics Institute (BGI) Huo-Yan Engineering Technology, Shenzhen, China; 3 Beijing Genomics Institute (BGI) Research, Shenzhen, China; 4 College of Traditional Chinese Medicine, Changchun University of Chinese Medicine, Changchun, China; 5 Beijing Genomics Institute (BGI) Research, Hangzhou, China; 6 Shenzhen Proof-of-Concept Center of Digital Cytopathology, Beijing Genomics Institute (BGI) Research, Shenzhen, China

**Keywords:** antibody responses, single-cell transcriptome sequencing, COVID-19 vaccine, vaccine effectiveness, antibody decline phase

## Abstract

**Introduction:**

Variability in antibody responses among individuals following vaccination is a common phenomenon. This study employs single-cell transcriptome sequencing to characterize the transcriptomic features underlying these variations during the antibody decline phase after inactivated COVID-19 vaccination.

**Methods:**

Sixty-five healthy volunteers received two doses of BBIBP-CorV. Antibody levels were measured 109–140 days post-vaccination. From these, 15 samples representing low, median, and high antibody titers were selected for PBMC single-cell RNA sequencing.

**Results:**

Re-analysis of antibody kinetics revealed that titers during the decline phase are most strongly associated with long-term persistence. Differentially expressed genes were enriched in immune pathways including lymphocyte activation, antigen presentation (MHC-I and MHC-II), and interferon signaling. Significant variation in HLA genes, particularly HLA-B, was observed across PBMC cell types. Cell-cell communication analysis further identified enhanced MHC-I signaling in high-titer groups, dominated by HLA-B interactions with CD8+ T cells.

**Discussion:**

These findings reveal a state of balanced immune alertness during the decline phase, providing insights into the cellular and molecular determinants of long-term humoral immunity and informing future vaccine design.

## Introduction

Vaccine efficacy is commonly assessed by measuring specific antibody levels post-vaccination, which are crucial for neutralizing pathogens and preventing disease. Studies confirm a positive correlation between antibody titers and protective capacity (Earle et al., 2021; Khoury et al., 2021; Gilbert et al., 2022; Khoury et al., 2023). However, significant inter-individual variability in antibody responses exists across COVID-19 vaccines, including mRNA and inactivated types, creating a spectrum from high to low responders (Wiedermann et al., 2016; Munro, 2021; Naaber et al., 2021; Lee and Linterman, 2022). This variability is attributed to both vaccine-related factors (e.g., antigen type, dosing schedule) and host-related factors (e.g., age, genetic background) (Pulendran et al., 2010; Wiedermann et al., 2016; Frommert et al., 2022). While these associations are well-established, the underlying cellular mechanisms driving differential antibody responses at the host level remain poorly characterized.

Single-cell transcriptome sequencing (scRNA-seq) has revolutionized the study of immune dynamics, including responses to COVID-19 vaccination (Zhang et al., 2020; Cao et al., 2021; Ren et al., 2021; Liu et al., 2022; Wang et al., 2022). Previous scRNA-seq work has characterized broad immune changes post-vaccination (Cohen et al., 2021; Oberhardt et al., 2021; Castro Dopico et al., 2022; Tong et al., 2023). However, the immune cell profiles associated with antibody titer strata remain uncharacterized, limiting our insight into the cellular basis of heterogeneous humoral responses.

Antibody kinetics follow distinct phases: lag, log, platform, and decline (Varona et al., 2021). While single-cell studies have largely focused on early phases (Wimmers et al., 2021; Mulè et al., 2024; Li et al., 2025), the decline phase (3–6 months post-vaccination) has gained attention for its role in long-term immunity (Kang et al., 2025; Simonis et al., 2025). This phase represents a transition to memory persistence, where short-lived plasma cells wane, and long-lived memory cells (e.g., memory B cells, T cells) dominate (Dan et al., 2021; Guo et al., 2024). Thus, the decline phase offers a unique window to assess the quality and durability of immune memory, which may correlate with protection against severe disease (Bar-On et al., 2021; McMahan et al., 2021; Wang et al., 2021).

To address these gaps, we aimed to define the single-cell transcriptomic profiles of PBMCs during the antibody decline phase after inactivated COVID-19 vaccination (BBIBP-CorV). By comparing individuals with low, median, and high antibody titers, we seek to identify immune cell subsets and transcriptional programs associated with antibody persistence, thereby elucidating mechanisms of long-term humoral immunity.

### Result 1: decline phase was more strongly associated with long-term antibody persistence than the platform phase

**Figure 1:**
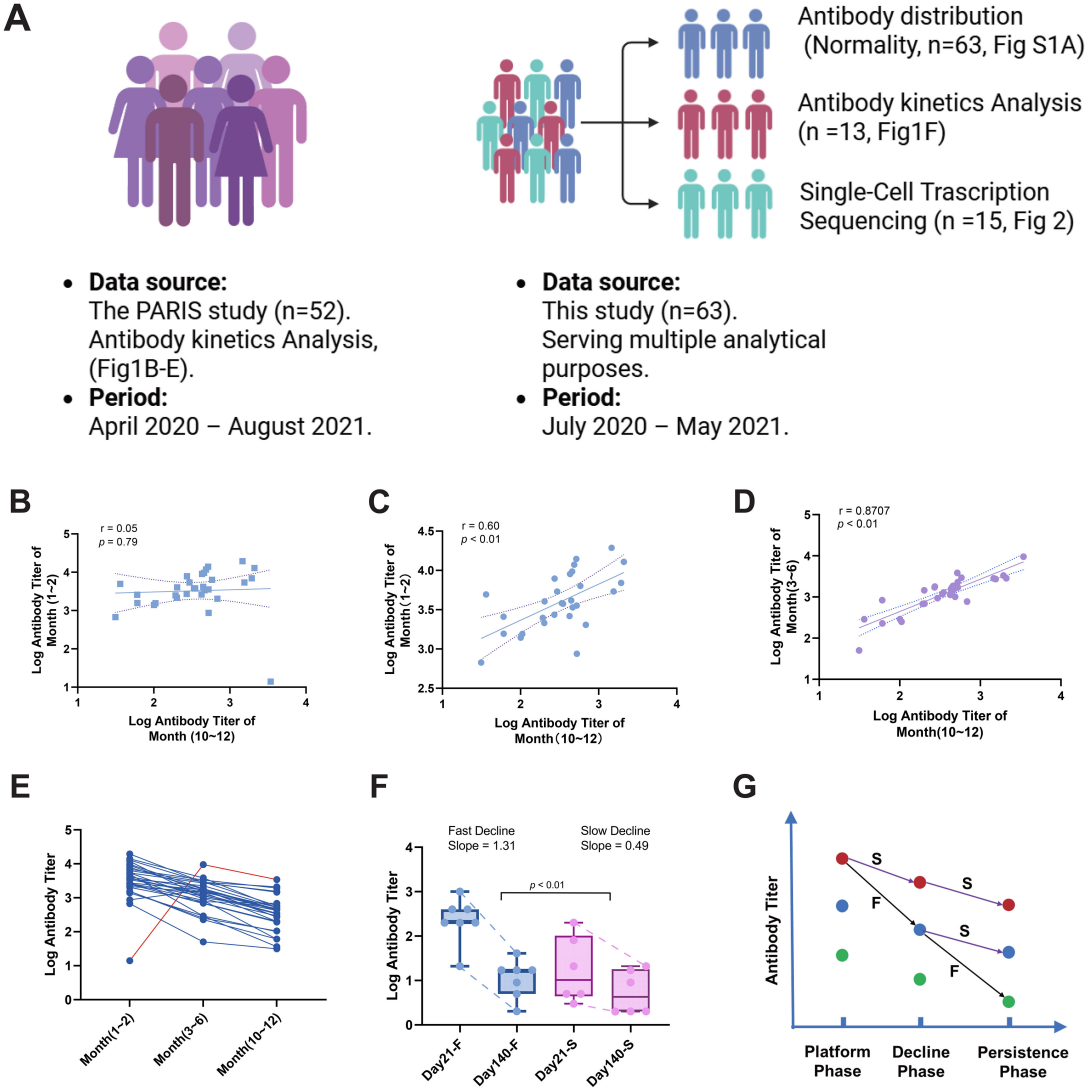
Antibody titer dynamics and correlation analysis following COVID-19 vaccination. **(A)** Schematic overview of data sources and analytical modules. Left: Data from the PARIS study (n=52; April 2020 – August 2021) used for antibody kinetics analysis (panels B–E).Right: Data from the present study (n=63; July 2020 – May 2021) used for multiple analytical purposes: antibody distribution and normality assessment (n=63; **Supplementary Figure S1A**), antibody kinetics (n=13; panel F), and single-cell transcriptomics (n=15; **Figure 2**). **(B)** Correlation of antibody titers between the platform phase (1–2 months) and persistence phase (10–12 months) using all data points. **(C)** Correlation after excluding an outlier from the platform vs. persistence phase analysis. **(D)** Correlation of antibody titers between the decline phase (3–6 months) and persistence phase. All panels display log-transformed antibody titers on both axes unless otherwise specified. **(E)** Identification of an outlier exhibiting a delayed antibody response, with lower titers during the platform phase that increased by the decline phase. **(F)** Comparison of antibody decline rates between the fast- and slow-declining groups (denoted as F and S, respectively). **(G)** Redistribution of antibody titer groups over time due to heterogeneous decay kinetics. The schematic illustrates how individuals with fast (red), intermediate (blue), and slow (green) decay rates change their relative positions. The abbreviations F and S denote Fast and Slow decline rates, respectively.

To investigate which post-vaccination antibody phase most effectively predicts long-term humoral immunity, we analyzed antibody kinetics using historic data from the PARIS study (Simon et al., 2022) and our independently collected dataset (**Figure 1A**). Antibody responses were stratified into three kinetic phases based on established decline patterns: the platform phase (1–2 months after the second dose), characterized by initially high antibody levels that begin a gradual decline; the decline phase (3–6 months), where a pronounced reduction in antibody titers occurs across most individuals; and the persistence phase (10–12 months), representing long-term antibody maintenance prior to the widespread influence of booster doses or emerging variants.

We then analyzed antibody titers from the platform and decline phases to determine which exhibited a stronger correlation with titers in the persistence phase. The analysis showed that the correlation between antibody titers in the decline phase and the persistence phase was significantly stronger than that between the platform phase and the persistence phase (**Figure 1B** vs. **1D**), a finding that held even after excluding an outlier (**Figure 1C**). This outlier (marked in **Figure 1E**) exhibited a ‘delayed antibody response,’ characterized by lower titers during the platform phase that increased by the decline phase.

We hypothesize that the observed variation in antibody titers across time points is attributable to individual differences in antibody decline rates. This is supported by the significant difference in decline rates between the fast- and slow-declining groups (p < 0.01, **Figure 1F**). These differing rates cause a redistribution of samples into different titer groups over time (**Figure 1G**). As a result, the decline phase exhibits less variability and a stronger correlation with the persistence phase, making it a superior predictor for assessing long-term antibody persistence differences among individuals.

Furthermore, the heterogeneity in antibody decline rates inherently reshuffles the relative ranking of individuals within the antibody titer distribution over time. As illustrated in **Figure 1G**, samples initially clustered in the high-titer group (red dots) during the platform phase subsequently diverged into intermediate- or low-titer groups (blue or green dots) during the decline phase, a redistribution driven by their disparate decay kinetics. Consequently, an individual’s antibody level at an early time point becomes a less reliable predictor of their later level due to this temporal reshuffling. This increased variability in titer trajectories over time directly explains the weaker correlation observed between the platform and persistence phases compared to that between the decline and persistence phases.

In summary, the analysis of historical antibody kinetic data revealed that antibody titers during the decline phase correlate more strongly with long-term persistence than those in the platform phase. This is likely due to the reduced inter-individual variability and greater stability of antibody levels during this phase, which minimizes noise and allows for a clearer assessment of an individual’s inherent immune response. Accordingly, antibody titer in the decline phase is proposed as a candidate metric for assessing differences in long-term humoral immunity among individuals.

### Result 2: increased proportions of activated CD4 T cells, DC and monocytes with rising antibody titers

**Figure 2:**
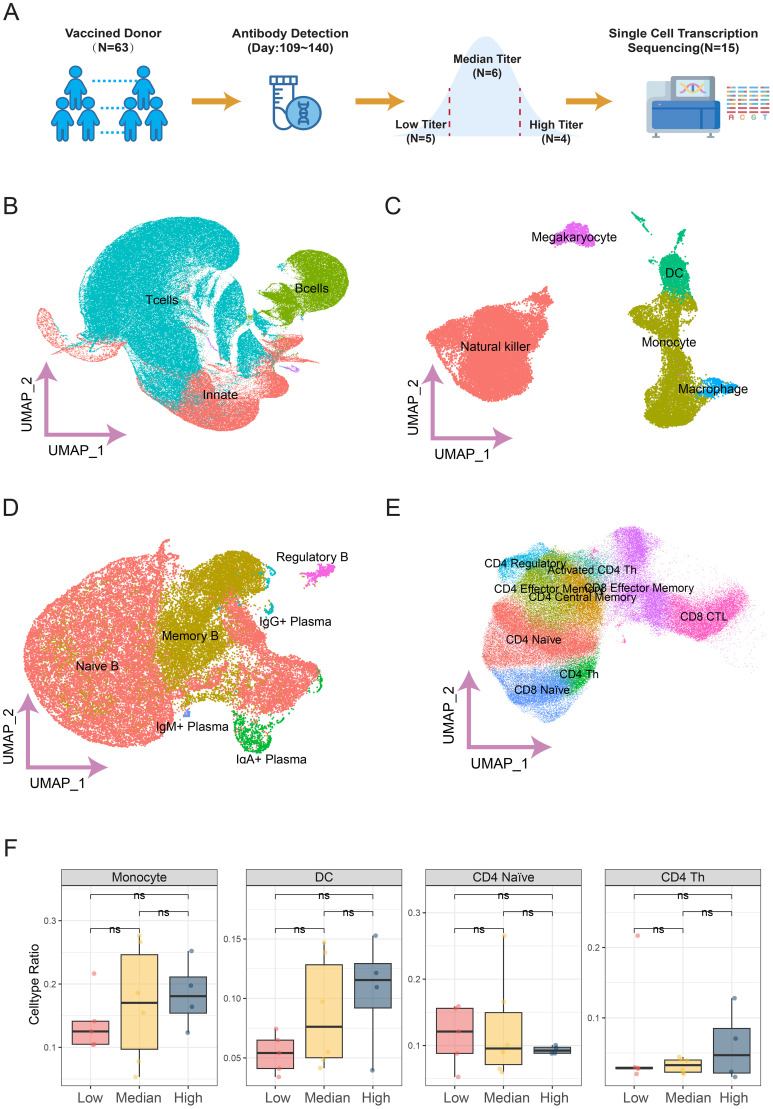
Single-cell transcriptome analysis of PBMCS from healthy donors after two SARS-CoV-2 vaccinations. Panel **(A)** illustrates the sample grouping and collection process. Panels **(B–E)** display UMAP plots that cluster various cell types, including Innate immune cells **(C)**, different B cell subpopulations **(D)**, and diverse T cell subpopulations **(E)**. Panel **(F)** provides a statistical analysis of cell proportions in relation to antibody titers, indicating which cell types show increased or decreased proportions with antibody levels, with the x-axis representing cell types and the y-axis showing their percentages.

To investigate differences in immune mechanisms during the antibody decline phase following two doses of the BBIBP-CorV vaccine, we initially selected 65 individuals from a historical cohort with measurable antibody titer data at 109 to 140 days post-vaccination. These individuals were stratified into three groups based on IgG level distribution: the highest 10th percentile as the high antibody group, the lowest 10th percentile as the low antibody group, and the remaining 80% as the medium antibody group (**Supplementary Figure S1**). Analysis of baseline characteristics confirmed that sex was balanced and that there were no significant differences in age across the titer groups (see **Supplementary Table S1**). From each group, 4 to 6 samples were randomly selected for single-cell transcriptome sequencing, resulting in a total of 15 samples included for subsequent analysis (**Figure 2A**).

Based on the sequencing transcriptome data of PBMCs from the selected samples, the total sequencing cells were classified into three main types: innate immune cells, B cells, and T cells, using a set of classic cell surface marker genes (see **Supplementary Table S2**). The cells were then further clustered, and subpopulations within these categories were identified based on highly variable genes and classic markers. After excluding ambiguous or non-classic cell types, 19 distinct cell subpopulations were successfully identified.

Overall, the proportions of identified cell types were consistent with traditional morphological estimates (**Supplementary Table S3**). For instance, previous studies indicate that T cells typically comprise 60% to 80% of PBMCs, while our analysis showed T cells accounted for 66.37%. Among B cells, most were naïve B cells (59.49%), followed by memory B cells (36.61%). Additionally, Monocytes, dendritic cells (DC), and macrophages belong to the myeloid lineage and share similar functional and origin characteristics, leading to closer clustering in gene expression profiles. In contrast, natural killer (NK) cells, which belong to the lymphoid lineage, were more distantly clustered.

We also analyzed differences in cell proportions across antibody groups and observed considerable variability among the samples (**Supplementary Figure S2**). While the proportions of monocytes, DC cells, and CD4 Th cells increased with higher antibody titers, CD4 Naive T cells showed a decrease (**Figure 2F**). However, these differences were not statistically significant, likely due to the limited sample size between groups.

### Result 3: characterization of differentially expressed genes in the decline phase

**Figure 3:**
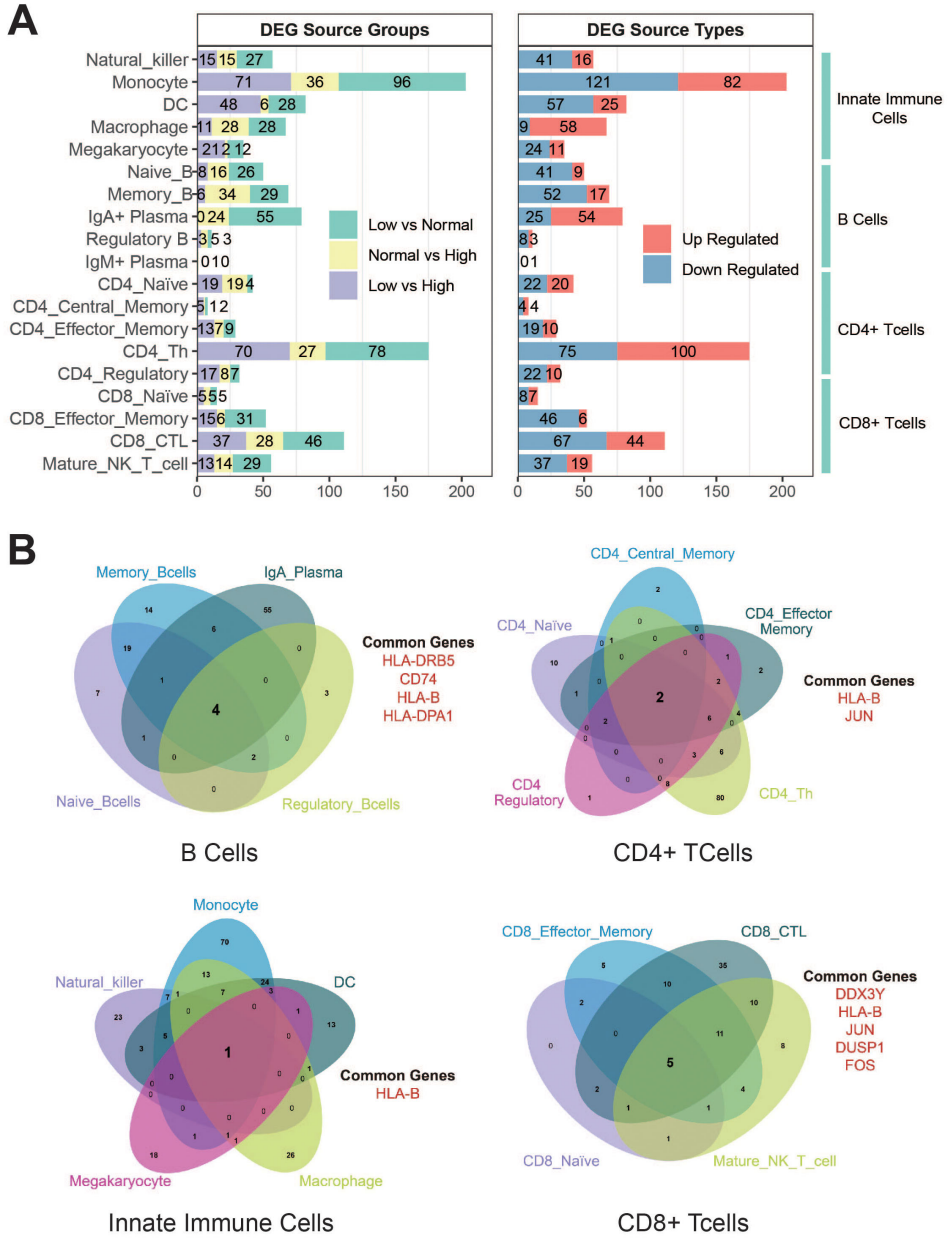
Differential expression genes (DEGs) affecting antibody titers across various cell types. **(A)** Differential gene expression across immune cell types. The left panel shows DEG counts from pairwise comparisons between antibody titer strata: Low vs. Median (light green), Median vs. High (light yellow), and Low vs. High (light purple). The right panel categorizes the total DEGs for each cell type as up-regulated (red; log_2_FC > 0.3) or down-regulated (blue; log_2_FC < -0.3). DEGs were defined using an adjusted p-value < 0.01. **(B)** Venn diagrams depicting the overlap of DEGs among subsets within major immune cell lineages, including innate immune cells, B cells, CD4^+^ T cells, and CD8^+^ T cells. Shared genes across subsets suggest core functional adaptations within each lineage during the antibody decline phase.

To delineate the transcriptomic adaptation of immune cells during the antibody decline phase, we performed differential gene expression (DGE) analysis across groups stratified by antibody levels (low vs. median, and median vs. high). DEGs were identified through pairwise comparisons between these adjacent titer groups, with the higher-titer group serving as the baseline for determining up- or down-regulation. Genes were considered significantly differentially expressed using a threshold of |log_2_(fold change)| > 0.3 (i.e., log_2_FC > 0.3 for up-regulated; log_2_FC < –0.3 for down-regulated) and an adjusted p-value < 0.01 (see Methods for details). Following this approach, monocytes, CD4^+^ Th cells, and CD8^+^ CTL cells exhibited the highest numbers of differentially expressed genes (DEGs) (203, 175, and 111, respectively), indicating that both innate immune cells and key adaptive effector populations undergo the most profound transcriptomic reprogramming, underscoring their central role in responding to waning antibody titers (**Figure 3A**). Significant changes were also observed across B cell subsets, consistent with their direct role in antibody production.

Notably, the number of differentially expressed genes (DEGs) was markedly higher in the Low vs. Median comparison than in the Median vs. High comparison (e.g., monocytes: 96 vs. 36; CD4^+^ Th cells: 78 vs. 27) (**Figure 3A**, left panel), indicating that the transcriptomic changes are most pronounced when antibody titers drop below the median level. We hypothesize that this median titer may represent a critical biological threshold, beyond which more substantial immune adaptations are required. This is supported by the bias toward transcriptional suppression in most cell types and activation in macrophages and CD4^+^ Th cells, collectively depicting a state of balanced yet dynamic immune adaptation during the antibody decline phase.

In contrast, most immune cell types—including monocytes, CD8^+^ CTL cells, dendritic cells (DCs), and natural killer (NK) cells—exhibited a pronounced bias toward down-regulated DEGs, reflecting broad transcriptional suppression during the antibody decline phase (**Figure 3A**, right panel). These subsets consistently showed more down-regulated than up-regulated DEGs. Conversely, macrophages displayed strong transcriptional activation, with a substantial predominance of up-regulated DEGs (58 vs. 9). Similarly, CD4^+^ Th cells also exhibited a bias toward up-regulation (100 vs. 75), consistent with a highly active functional state.

Venn diagram analysis further revealed functionally coherent patterns through shared DEGs across cell lineages (**Figure 3B**). B cell subsets shared genes associated with the antigen presentation pathway via MHC class II (e.g., HLA-DRB5, HLA-DPA1, CD74), indicating a conserved or enhanced antigen-presenting capacity. CD8^+^ T and cytotoxic subsets shared immediate-early genes such as JUN and FOS, consistent with a state of cellular alertness. Notably, HLA-B (an MHC class I molecule) was the only shared gene among innate immune cells, suggesting a unified role in sustained MHC-I-mediated surveillance.

In summary, these findings depict a dynamic yet balanced immune adaptation during the antibody decline phase, characterized by a critical response threshold and coordinated functional reprogramming across major immune cell lineages.

### Results 4: antibody decline phase is associated with a characteristic transcriptomic state of the immune system

**Figure 4:**
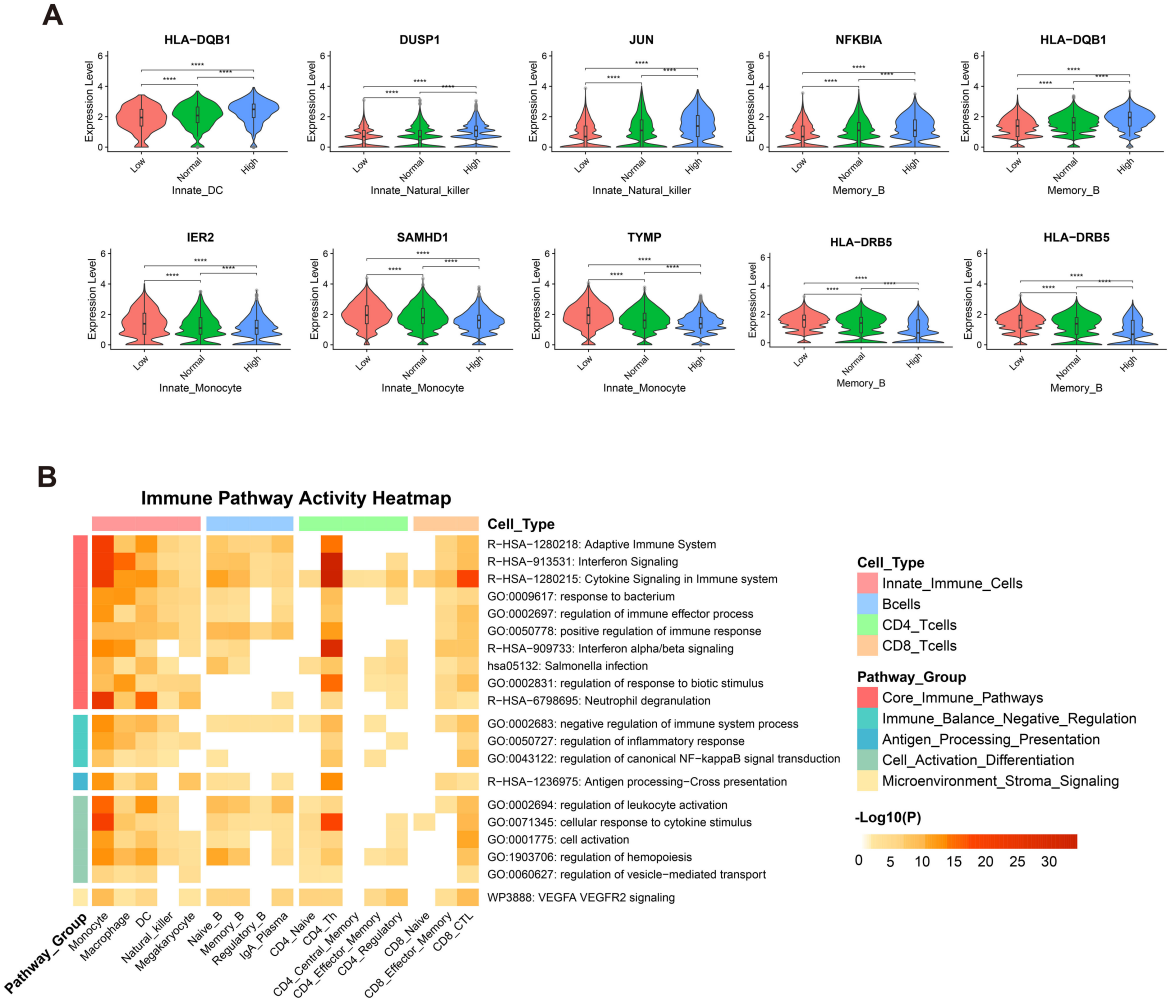
Enrichment map of differential genes influencing antibody expression in various cells. **(A)** Visualization of continuously up- or down-regulated genes during the antibody decline phase. The upper panel displays genes with consistent up-regulation, while the lower panel shows down-regulated genes, across low, median, and high antibody titer groups. Genes were identified through differential expression analysis (adjusted p-value < 0.01). A complete list of these genes is provided in **Supplementary Table S5**. **(B)** Heatmap illustrating the enrichment of biological terms that influence antibody expression across different cell types. The x-axis lists the categories of cells, while the y-axis lists representative biological terms from similar term clusters. The color intensity within each cell of the heatmap signifies the level of enrichment significance, with gray indicating a lack of enrichment for that pathway in the specified cell type.

We identified 26 genes exhibiting concordant upregulation or downregulation across all immune cell types, meaning their expression changes were significant in both the Low vs. Median and Median vs. High antibody-level comparisons during the decline phase (**Figure 4A**; **Supplementary Table S5**). The upregulated genes were primarily associated with immune negative regulation (e.g., NFKBIA in naïve and memory B cells), antigen presentation (e.g., HLA-DQB1 in B and dendritic cells), and cellular alertness (e.g., FOS/JUN in NK and cytotoxic T cells). The downregulated genes, including interferon-stimulated genes such as GBP1 and STAT1 in CD4^+^ Th cells, indicated an attenuation of acute antiviral responses, reflecting a transition from vaccine-induced activation toward immune homeostasis.

To systematically decipher the biological functions and coordinated regulation behind these gene changes, we performed cross-cell-type pathway enrichment analysis using Metascape (Zhou et al., 2019), integrating multiple databases. It is important to note that this over-representation analysis (ORA) identifies pathways enriched with differentially expressed genes but does not inherently indicate the direction (up- or down-regulation) of the entire pathway. This analysis revealed 239 significantly enriched pathways, summarized into 20 functional clusters (**Supplementary Tables S6, S7**).

Pathway enrichment analysis revealed that during the antibody decline phase, the immune system does not enter a quiescent state but undergoes significant transcriptomic reprogramming, with particularly prominent changes in core immune effector cells including CD4^+^ T cells, monocytes, and cytotoxic T cells (**Figure 4B**). In these cells, essential immune response functions such as interferon and cytokine signaling pathways (R-HSA-913531 and R-HSA-1280215; NES < -1, **Supplementary Table S8**, **Supplementary Figure S3**), indicating preserved effector capabilities and a reduced state of alertness. Crucially, negative immune regulatory pathways (GO:0002683) were also up-regulated in these populations, demonstrating a precisely balanced immune response that maintains baseline defensive functions while suppressing excessive inflammation and autoimmune damage, consistent with an “alert but not overactive” immune homeostasis hypothesis.

Additionally, the enrichment of antigen processing and presentation pathways (R-HSA-1236975) suggests sustained operation of immune surveillance mechanisms, providing a molecular basis for long-term immunological memory. The broad up-regulated of pathways related to cell activation and hematopoietic differentiation (GO:0002694, GO:0001775) further indicates that the immune system maintains active functional preservation and renewal.

In summary, the immune state during the antibody decline phase can be characterized as a multi-cellular coordinated, balanced, and sustainable alert state, supported by robust core effector functions, precise negative feedback regulation, continuous antigen surveillance, and active cellular differentiation and activation.

### Result 5: regulation of lymphocyte activation is a central pathway regulating antibody titers during the decline phase

**Figure 5:**
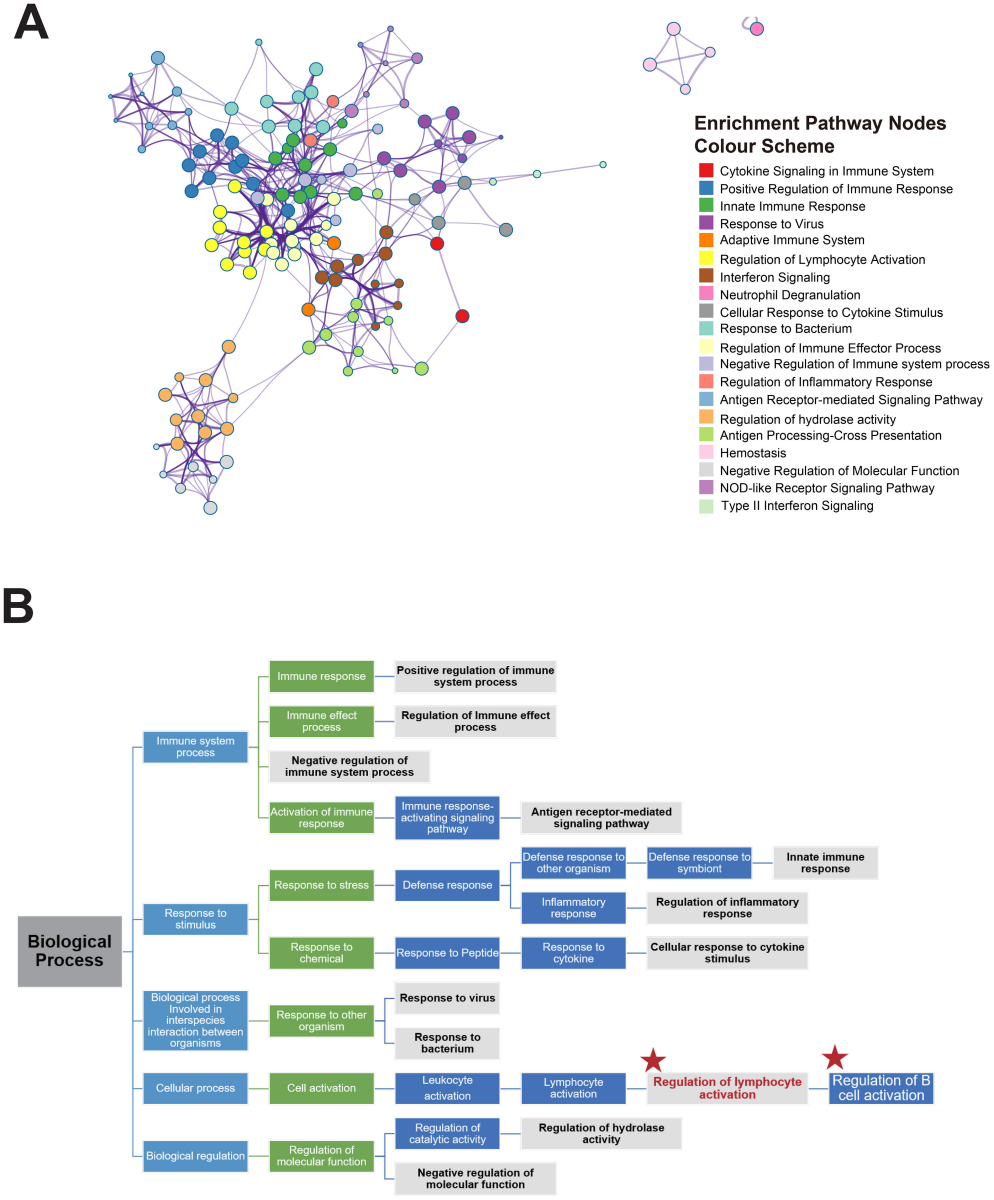
Gene pathway interactions and ancestor diagram of GO pathways. **(A)** Gene pathway interaction network. Node colors represent distinct pathways, and edge thickness corresponds to the degree of similarity between pathways. **(B)** Directed acyclic graph (DAG) of significantly enriched GO terms. Pathways significantly enriched during the antibody decline phase are highlighted with a gray background and bold font. The central pathway and its child pathways are marked by red asterisks.

To elucidate the interconnections between key biological processes during the antibody decline phase, we performed a network analysis of the significantly enriched pathways. These relationships are illustrated in **Figure 5A**, where GO:0051249 (regulation of lymphocyte activation, green dots) stands out as central and highly significant (**Figure 5A**, **Supplementary Table S7**). This pathway plays a crucial role in regulating lymphocyte activation, including that of B cells, and is directly linked to other key pathways such as *GO*:0001775 Cell Activation, yellow dots), R-HSA-913531 (interferon signaling, purple dots), and GO:0002697 (Regulation of Immune Effector Process, gray dots). Additionally, GO:0050864 (regulation of B cell activation, **Figure 5B**, red asterisks) is a direct subcategory of GO:0051249 (regulation of lymphocyte activation). This emphasizes the central role of lymphocyte activation regulation in the pathway enrichment network, which highlights important pathways influencing antibody titers during the decline phase following vaccination. The results show that this pathway not only governs lymphocyte activation but also interacts with other crucial immune response pathways, including those involved in innate immunity and cytokine responses.

In the broader diagram of the enriched GO pathways (**Figure 5B**), we observe that these pathways primarily relate to the immune system, stimulus responses, cellular processes, and biological regulation. Of the 12 primary GO pathways identified, half are associated with regulatory functions, such as immune response regulation, lymphocyte activation regulation, immune effect regulation, inflammatory response regulation, hydrolase regulation, and negative regulation of molecular function (**Supplementary Table S7**). This raises questions about potential commonalities among these regulatory pathways in their role in influencing antibody titers during the decline phase post-vaccination, which merit further investigation.

### Result 6: HLA-B gene variation highlights divergent antigen recognition capacity across antibody titer groups

**Figure 6:**
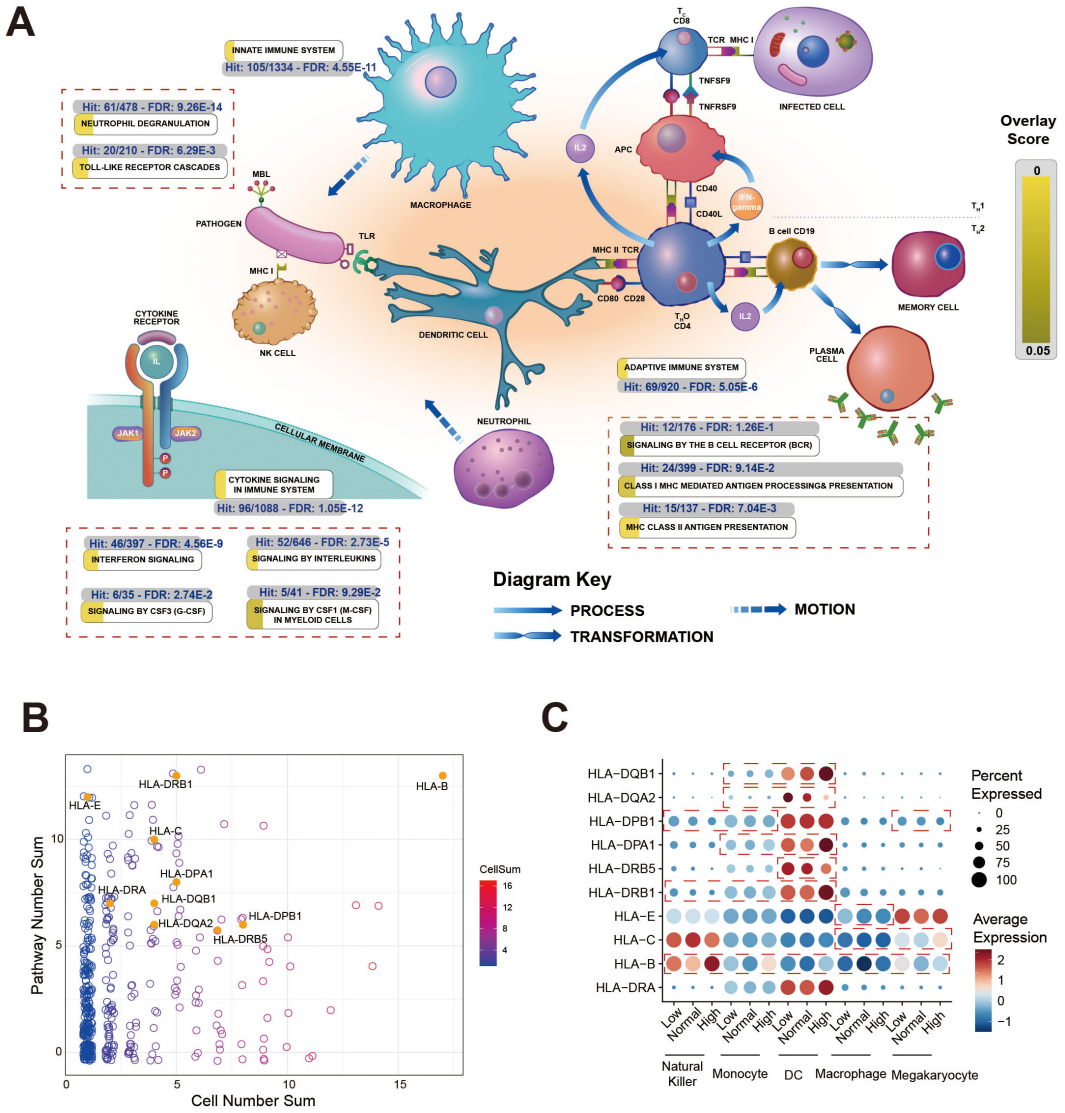
Immune landscape during the antibody decline phase. **(A)** Adapted Reactome immune system pathway diagram. Cellular components and interactions of the innate and adaptive immune system are shown. Pathways that were significantly enriched in our dataset (based on the combined set of DEGs from all inter-group comparisons) are highlighted. The color intensity of the overlay corresponds to the statistical significance of the enrichment, represented as -log_10_FDR. Specifically, the color scale ranges from light yellow (less significant) to dark green (most significant), as indicated by the scale bar. **(B)** Scatter plot of gene prevalence across cell types and pathways. Each point represents a gene. The x-axis (Cell Number Sum) indicates the number of cell types in which the gene was differentially expressed. The y-axis (Pathway Number Sum) indicates the number of enriched pathways associated with that gene. Point color corresponds to the cumulative expression level (CellSum) across cell types, with HLA genes labeled. **(C)** Expression profile of HLA genes across immune cell types. Rows represent individual HLA genes, columns represent cell types stratified by antibody titer groups (Low, Median, High). The heatmap color scale (right) indicates mean gene expression level (red: high, blue: low). The size of the circle within each cell represents the proportion of cells within that type expressing the gene (0-100%).

To gain a global perspective on the immune processes associated with antibody titer variation, we performed a pathway analysis using the Reactome framework on the combined set of differentially expressed genes (DEGs) from all inter-group comparisons. Specifically, 43.1% of differentially expressed genes (DEGs) mapped to immune system pathways, while 32.9% were involved in signaling pathways. Three major immune modules were particularly prominent: cytokine signaling (FDR: 1.05×10^-^¹²), innate immune system (FDR: 4.55×10^-^¹¹), and adaptive immune response (FDR: 5.05×10^-6^) (**Figure 6A**). Within the cytokine signaling module, the interferon-gamma (IFN-γ) pathway emerged as a central node. IFN-γ enhances B cell antigen response, promotes class switching, and supports memory B cell survival, suggesting that vaccine-induced IFN-γ signaling helps sustain long-term humoral immunity.

At the gene level, HLA-B exhibited the most pronounced variation across antibody titer groups. It was identified as a DEG in 17 immune cell types and appeared in 7 out of 14 representative pathways (**Supplementary Table S6**). Its expression differed significantly across cell types and antibody concentration levels, reflecting high inter-individual variability (**Figure 6B**). As a classical MHC-I gene, HLA-B mediates antigen presentation to CD8^+^ T cells, which indirectly influences antibody responses via cytokine secretion (e.g., IFN-γ). This aligns with the observed enrichment of the IFN-γ pathway, suggesting that HLA-B variation is associated with antibody persistence through T cell mediated mechanisms rather than direct B cell activation.

Beyond HLA-B, multiple HLA genes were recurrently identified within the DEG set across cell types, including MHC-I genes (HLA-B, HLA-C, HLA-E) and MHC-II genes (HLA-DP, -DQ, -DR) (**Figures 6B, C**). Notably, dendritic cells showed elevated expression of HLA class II genes (**Figures 6B, C**). Together, these data demonstrate a broad and pronounced pattern of differential expression across the HLA gene family in relation to antibody titer groups.

### Result 7: cell-cell communication networks underlying heterogeneous antibody maintenance

To understand how the genetic variations in antigen presentation machinery (e.g., HLA-B) may translate into functional immune coordination, we next investigated the cell-cell communication networks across antibody titer groups. Our analysis revealed distinct interaction patterns during the antibody decline phase (**Supplementary Figure S4A**). The communication network demonstrated extensive connectivity without dominant specific cell-cell interactions (**Supplementary Figure S4B**). Comparative analysis between high and low antibody titer groups showed significantly more enhanced interactions than diminished interactions (**Supplementary Figure S4B**), indicating a positive association between communication intensity and antibody maintenance capacity.

**Figure 7:**
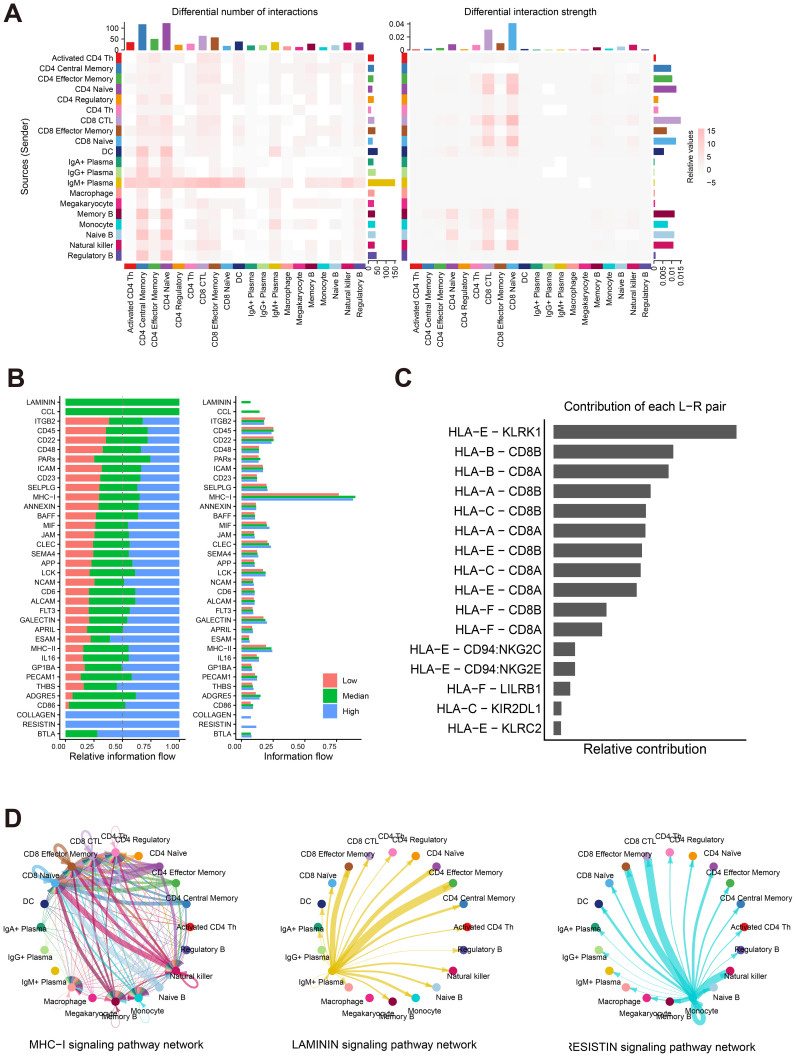
Cell–cell communication networks and signaling pathway activity across antibody titer groups. **(A)** Heatmaps depicting the number (left) and strength (right) of differential interactions among major cell types. **(B)** Bar plots showing the relative information flow of signaling pathways across low (red), median (green), and high (blue) antibody titer groups.(Left) Stacked bar plot displaying the normalized relative contribution of each signaling pathway to the total information flow within each titer group.(Right) Non-stacked bar plot showing the absolute information flow strength of each signaling pathway across titer groups. **(C)** Bar plot indicating the relative contribution of ligand–receptor pairs to signaling pathway activity. **(D)** Communication networks of key signaling pathways: MHC-I (left), LAMININ (middle), and RESISTIN (right). Nodes represent cell types; edges represent interaction strengths.

Evaluation of cellular communication roles identified memory B cells, CD4 naive T cells, and CD8 CTL cells as the most active signal sources based on interaction strength (**Figure 7A** right panel). While IgM+ plasma cells showed frequent signaling activity, their communication strength was comparatively limited (**Figure 7A** left panel). CD8 CTL and CD8 naive T cells functioned as primary signal receivers, exhibiting significantly higher signal reception intensity than other cell populations (**Figure 7A** right panel).

Analysis of pathway-level information flow revealed that the MHC-I signaling pathway demonstrated predominant and stable activity across titer groups, evidenced by its high strength (**Figure 7B**, right) and consistent relative contribution (**Figure 7B**, left). This activity was predominantly driven by the HLA-E – KLRK1 pair, with substantial contributions from HLA-B – CD8B and HLA-B – CD8A (**Figure 7C**).

Group-specific communication patterns revealed distinct pathway activation: MHC-I-related communication enhancement was widely present across multiple cell types (**Figure 7D** left panel); LAMININ signaling was exclusively elevated in the median titer group and predominantly originated from IgM+ plasma cells (**Figure 7D** middle panel), while RESISTIN signaling was specifically enhanced in the high titer group and mainly derived from monocyte populations (**Figure 7D** right panel).

## Discussion

Our study identifies the antibody decline phase (3–6 months post-vaccination) as a superior predictor of long-term humoral immunity compared to the platform phase. This finding, consistent with established kinetic models (Varona et al., 2021; Srivastava et al., 2024), is driven by individual heterogeneity in decay rates—a factor often overlooked in population-level averages (Xia et al., 2021; Poehler et al., 2022). The stronger correlation is consistent with the waning of short-lived plasma cells, which may unmask the influence of the intrinsic quality of the memory compartment on sustained antibody levels (Cohen et al., 2021; Dan et al., 2021). This paradigm is supported by recent studies across vaccine platforms. For instance, research on mRNA vaccines also highlights the critical importance of the decay phase in determining neutralizing antibody breadth and durability against variants (Israel et al., 2021; Kang et al., 2025). Our analysis of an inactivated vaccine platform confirms that this phase is a universal window into the establishment of lasting immune memory.

A central finding of our deep immunological profiling is the pivotal role of the MHC-I gene HLA-B. We identified significant expression variation of HLA-B across 17 immune cell types, and it identified as a shared node in 7 core pathways. While HLA genes are established modifiers of vaccine response (Martin et al., 2023; Tong et al., 2023), our data uniquely pinpoint HLA-B as the most dynamically variable MHC-I gene during the decline phase. We hypothesize that its polymorphisms are associated with variations in antigen presentation to CD8^+^ T cells. The resulting CD8^+^ T cell responses (e.g., IFN-γ production) may indirectly shape the overall immune environment (Hoekstra et al., 2024), potentially influencing CD4^+^ T cell helps for B cells and antibody persistence. These patterns collectively highlight that natural polymorphisms in HLA genes, particularly HLA-B, are linked to heterogeneity in antigen recognition and immune coordination, which coincides with the divergence in antibody maintenance during the decline phase post-vaccination. This genetic influence manifests functionally in cell-cell communication networks. Our CellChat analysis revealed that MHC-I signaling dominated intercellular communication across all titer groups, primarily driven by enhanced interactions between HLA-B and its receptors (CD8A/B) on CD8^+^ T cells. These results delineate a communication network architecture where enhanced MHC-I-mediated signaling, particularly involving CD8^+^ T cell interactions, is associated with superior antibody maintenance. The communication patterns observed complement the HLA-B variation findings, demonstrating how genetic polymorphisms in antigen presentation machinery may coincide with cell-cell communication networks and differential antibody maintenance capabilities among individuals. This aligns with non-human primate studies identifying CD8^+^ T cells as correlates of protection (McMahan et al., 2021) and echoes findings from mRNA vaccine studies where early CD8^+^ T cell responses were linked to long-term durability (Nouanesengsy et al., ).

Our findings suggest that HLA-B-driven coordination may underlie a global immune state of dynamic equilibrium during the decline phase. This state was characterized by the concurrent enrichment of both activating (e.g., cell activation) and regulatory pathways. We propose that this molecular pattern could represent a potential adaptive equilibrium, which may be a feature of the sustained immune surveillance observed in high-titer individuals. Alternatively, it might reflect a transitional state during the normal contraction of the immune response or a dynamic recalibration of signaling networks (Mulè et al., 2024). This transcriptional reprogramming was achieved primarily within stable cell populations rather than through changes in cellular composition. Key effector cells like monocytes, CD4^+^ Th, and CD8^+^ CTL cells assumed distinct functional states across titer groups despite conserved frequencies. This observation supports the notion that immune durability hinge on functional plasticity, a notion supported by a growing body of literature on SARS-CoV-2 vaccination. Studies on both mRNA and adenovirus-vectored vaccines have demonstrated that transcriptional states of innate immune cells and antigen-specific T cells are stronger predictors of neutralizing antibody responses than cell frequency alone (Zhou et al., 2023; Fujitani et al., 2025). Our findings in the context of an inactivated vaccine extend this principle, advocating for vaccine assessment strategies that move beyond simple cell counts or peak titers to incorporate transcriptomic states. This is further reinforced by studies linking specific early cellular responses to antibody outcomes. For example, one study employing mass cytometry analyzed immune populations after administration of the mRNA vaccine BNT162b2, finding a correlation between a specific CD4+ ICOS+ CD38+ cell subset and IgG production (r=0.68, p=0.03) (Kramer et al., (2022)). Our work complements this by defining the sustained transcriptional programs that underpin durability long after the initial response.

A critical question is how our findings, derived from an inactivated vaccine, relate to other platforms and the evolving pandemic landscape. A recent comparative analysis underscores that humoral waning kinetics are indeed dictated by both disease severity and vaccine platform (Tong et al., 2024). For instance, mRNA vaccines induce potent but rapidly waning responses against Omicron, while infection-acquired immunity shows distinct durability patterns based on severity. Importantly, that study found that boosting, regardless of platform, can broaden and stabilize responses. This resonates with our framework: the “quality” of the memory compartment we describe—orchestrated by HLA genetics and transcriptomic reprogramming—may be the fundamental determinant that different platforms shape with different kinetics. Given the importance of the antibody decay phase that is being discussed, how do our findings translate to the current status of COVID-19, with all the concern pertaining variant emergence and SARS-CoV-2 escape mutants? In the context of immune-evasive variants like Omicron, where neutralizing antibody titers are a less reliable correlate against infection, the robust, balanced memory compartment we describe—characterized by sustained MHC-I signaling and a poised transcriptional state—likely becomes even more critical. This machinery is essential for mounting cross-reactive T-cell immunity and recalled B-cell responses, which are key for protection against severe disease from variants (Simonis et al., 2025). Thus, while the exact antibody titer against a specific variant may be less informative, the underlying quality of the memory compartment, which we mechanistically link to HLA-B and transcriptomic reprogramming, may be a more durable and variant-resistant correlate of long-term protection.

Our study has limitations. The analysis of PBMCs excludes key players in long-term immunity residing in bone marrow and lymphoid tissues. The modest scRNA-seq sample size (n=15) may limit power for rare subsets. The focus on an inactivated vaccine warrants caution in generalizing to other platforms, though our discussion above provides a framework for comparison. Finally, our bioinformatic insights require functional validation. Future work should include *in vitro* co-culture assays to test the functional impact of HLA-B variants on T-cell help for B cells, flow cytometric validation of T-cell states, and comparative studies across vaccine platforms to distinguish universal from mechanism-specific correlates of durability. Such efforts will be essential to translate these findings into improved vaccine strategies capable of inducing robust, variant-resilient immunity on a global scale.

## Methods

### Study subjects

Healthy adult individuals who had received the BBIBP-CorV COVID-19 vaccine were recruited as study subjects. In order to ensure data reliability and consistency, the following inclusion criteria were established: 1) participants with no prior SARS-CoV-2 infection who had completed the two-dose regimen of the inactivated COVID-19 vaccine; 2) the interval between the initial and booster doses was 3 to 4 weeks; 3) the final vaccination occurred between July 15, 2020, and August 30, 2020. Details including vaccination dates, gender, and age were recorded for each participant, with blood samples collected 21 days post-second dose and between 109 to 140 days post-vaccination to assess serum IgG antibody levels. Blood collection took place at BGI YouKang Clinic. All participants provided informed consent, and the study was approved by the BGI Institutional Review Board (approval number BGI-IRB 20161).

### Antibody detection

The SARS-CoV-2 RBD/S antibody detection kit (HWTS-RT055A, Macro & Micro-Test) was used to identify SARS-COV-2 specific IgG antibodies. This kit operates on a double-antigen sandwich enzyme-linked immunosorbent assay principle, with the SARS-CoV-2 RBD antigen initially coated on the microplate. Following this, blood samples are diluted in gradients and added to the microplate, then incubated with an enzyme-labeled antigen. In the presence of antibodies against the SARS-CoV-2 S protein RBD region, a “coated RBD antigen-SARS-CoV-2 antibody-enzyme-labeled RBD antigen” complex is formed. Addition of a chromogenic substrate leads to the enzyme catalyzing the production of a blue substrate. The reaction is halted, resulting in the substrate turning yellow. If RBD or S antibodies are absent in the sample, no color change occurs. The ELISA reader measures the OD value of the reaction, and positivity or negativity is determined based on a predefined cutoff value. The reciprocal of the dilution at which the OD falls below the critical reference value corresponds to the antibody titer in the serum.

### Peripheral blood mononuclear cell collection

For each participant, Peripheral blood samples (3 mL) were collected into EDTA anticoagulant tubes and gently mixed by inverting 4–6 times. The whole blood was then diluted with 3 mL of phosphate-buffered saline (PBS) and transferred to a 15 mL centrifuge tube. Using Ficoll-Paque Plus (Sigma Aldrich) solution, PBMCs were isolated following standard density gradient centrifugation methods. Cell collection and counting were performed using the Cellaca MX high-throughput cell counter (Nexcelom Bioscience). The isolated PBMCs were resuspended in a freezing medium consisting of 90% fetal bovine serum (FBS, HyClone) and 10% DMSO, followed by freezing at -80°C for 24 hours using Nalgene^®^ Mr. Frosty Cryo 1°C freezing containers (Thermo Fisher Scientific) before long-term storage in liquid nitrogen. All procedures were carried out under sterile conditions.

### Single-cell suspension preparation

Frozen PBMC storage vials were rapidly thawed in a 37°C water bath for approximately 2 minutes until only a small ice crystal remained. Thawed PBMCs were quenched with 4 mL of pre-warmed 1X phosphate-buffered saline (PBS, Thermo Fisher Scientific) at 37°C and supplemented with 10% FBS. The mixture was centrifuged at 500 x g for 10 minutes at room temperature. The supernatant was removed, and the cell pellet was resuspended in 3 mL of 1X PBS containing 0.04% bovine serum albumin (BSA, Sangon Biotech).

The resuspended cells were filtered through a 40 μm cell strainer (Falcon) and then subjected to centrifugation. Following the manufacturer’s protocol, dead cells were removed using magnetic bead purification (Miltenyi Biotech) before proceeding to single-cell RNA sequencing (scRNA-seq). The cells were resuspended in a cell resuspension buffer to achieve a concentration of 1000 cells/μL.

### Library construction and single-cell RNA-seq

We prepared scRNA-seq libraries using the DNBelab C single-cell library preparation kit (MGI, #1000021082) following previously reported methods. Briefly, cells were resuspended in a PBS solution containing 0.04% BSA and filtered through a 40μm cell strainer. The concentration of the cell suspension was recorded and measured for the use of DNBelab C single-cell library preparation kit (MGI, #1000021082) to generate droplets. After collecting the mixed droplets, emulsion breaking was performed, followed by reverse transcription, cDNA and oligonucleotide amplification, and product filtration. Oligonucleotide products were then barcode-labeled for PCR to create oligonucleotide circular libraries, while cDNA products were prepared into single-stranded DNA libraries. Finally, sequencing was conducted at the China National GeneBank (Shenzhen) using the DNBSEQ-T1 sequencer with a read length of 30bp for reads1 and 100bp for reads2.

### Single-cell RNA-seq data processing

The raw sequencing reads of DNBSEQ-T1 were filtered and demultiplexed by PISA (v.0.2) (https://github.com/shiquan/PISA). The reads were aligned to the human genome (GRCh38_release95) using STAR (v.2.7.4a) (Dobin et al., 2013) and sorted using Sambamba (v.0.7.0) (Tarasov et al., 2015). Doublet and contaminant cells were rigorously filtered based on clustering in the UMAP embedding space, unmarked expression, and estimation using DoubletFinder (McGinnis et al., 2019). Cells with feature counts below 200 or above 4000 (filtered out) and cells with mitochondrial UMI counts exceeding 10% (filtered out) were removed from the gene-cell matrix. Prior to filtering, the median gene count per cell across all cells was 3628 [IQR 2118-5497], and the median feature count was 1222 [IQR 848-1625]. After filtering, the median gene count per cell across all cells was 3768 [IQR 2287–5600], and the median feature count was 1253 [IQR 890–1645]. The SCTransform function in Seurat (version 4.1.1) was performed to normalize the data for further clustering purposes.

### Cell clustering, annotation, and DEGs identify

PCA and UMAP were used for dimensionality reduction in Seurat (version 4.1.1) (Hao et al., 2021). Based on markersets in the **Supplementary Table S2**, the first 20 principal components were utilized for UMAP projection and clustering analysis to differentiate innate immune cells, B cells, and T cells into three clusters. Subsequently, the Innate cells subtype marker set (dim=10, resolution=0.2), B cells subtype marker set (dim=15, resolution=0.2), T cell subtype marker set (dim=14, resolution=0.4) were used to identify subtypes within the three clusters. Following subtype identification, the barcode information from each cell-id was used to mapped back to the original three-cluster plot to visualize the cellular subtypes. For differential gene identification, the Seurat FindAllMarkers function was employed to recognize differentially expressed genes between clusters, with the parameters set as pos = TRUE, min.pct = 0.25, logfc.threshold = 0.3, test.use = ‘wilcox’. Genes with an adjusted p-value < 0.01 were considered differentially expressed.

### Differential gene enrichment and protein interaction analysis

The enrichment analysis of the differentially expressed genes in the transcriptome was conducted using the Metascape software (Zhou et al., 2019). Specifically, the differential genes from various cell types were tabulated and uploaded to the Metascape online software, and the resulting data was further processed using default parameters. For the differential genes in different cell types, we utilized Venn diagrams, violin plots, and bar charts for visualization. The Venn diagram was created using the online software Eveen (Chen et al., 2021), the violin plot used the built-in function VlinPlot in Seurat, and the bar chart was generated using ggplot2 (Wickham H, 2016).

By calculating the proportion of enriched pathways across all cell types, the representative 20 pathways were categorized into three groups: Common Pathway, Innate Immunity and B-cell Dominance, and Innate Immunity Dominant. The selection of representative pathways was based on the significance of the p-value and the cell type proportion. The pathway enrichment heatmap was plotted using the pheatmap (version 1.0.12) package in R. The network enrichment diagram was directly produced by Metascape, while the petal diagram was drawn using Eveen.

The MCODE network diagram was created by downloading the network generated by Metascape and visualizing it in Cytoscape (Shannon et al., 2003). The bubble charts for the pathways involved in each MCODE were plotted using R package ggplot2 (Wickham H, 2016).

### Reactome enrichment analysis

Pathway enrichment analysis was performed using the Reactome framework via its online GeneList analysis module. The input consisted of a combined, non-redundant list of all differentially expressed genes (adjusted p-value < 0.01, |log_2_(fold change)| > 0.3) derived from all pairwise comparisons between antibody titer groups (High vs. Median, High vs. Low, and Median vs. Low).

### CellChat analysis

Cell-cell communication analysis was performed using CellChat (v1.6.0) (Inference and analysis of cell-cell communication using CellChat, 2025) with default parameters to infer intercellular signaling networks based on curated ligand-receptor interactions. The input data consisted of normalized gene expression matrices and cell-type annotations derived from Seurat clustering. Significant interactions were identified using a probability threshold of >0.01, with ligands/receptors required to be expressed in at least 10% of cells within a cluster. Differential communication analysis between antibody titer groups was conducted to identify enriched or diminished signaling pathways. Visualization of networks was implemented via CellChat’s built-in functions for circle plots and chord diagrams.

## Data availability statement

The data that support the findings of this study have been deposited into CNGB Sequence Archive (CNSA) of China National GeneBank DataBase (CNGBdb) with accession number CNP0006517.
