# Supplementary material for: Single-cell transcriptome profile during the antibody decline phase following inactivated COVID-19 vaccination
Source: Front Cell Infect Microbiol. 2025 Dec 2;15:1715387. doi: 10.3389/fcimb.2025.1715387 (PMC12705540; doi:10.3389/fcimb.2025.1715387)
Supplement: Supplementary file 1 [file DataSheet1.pdf]

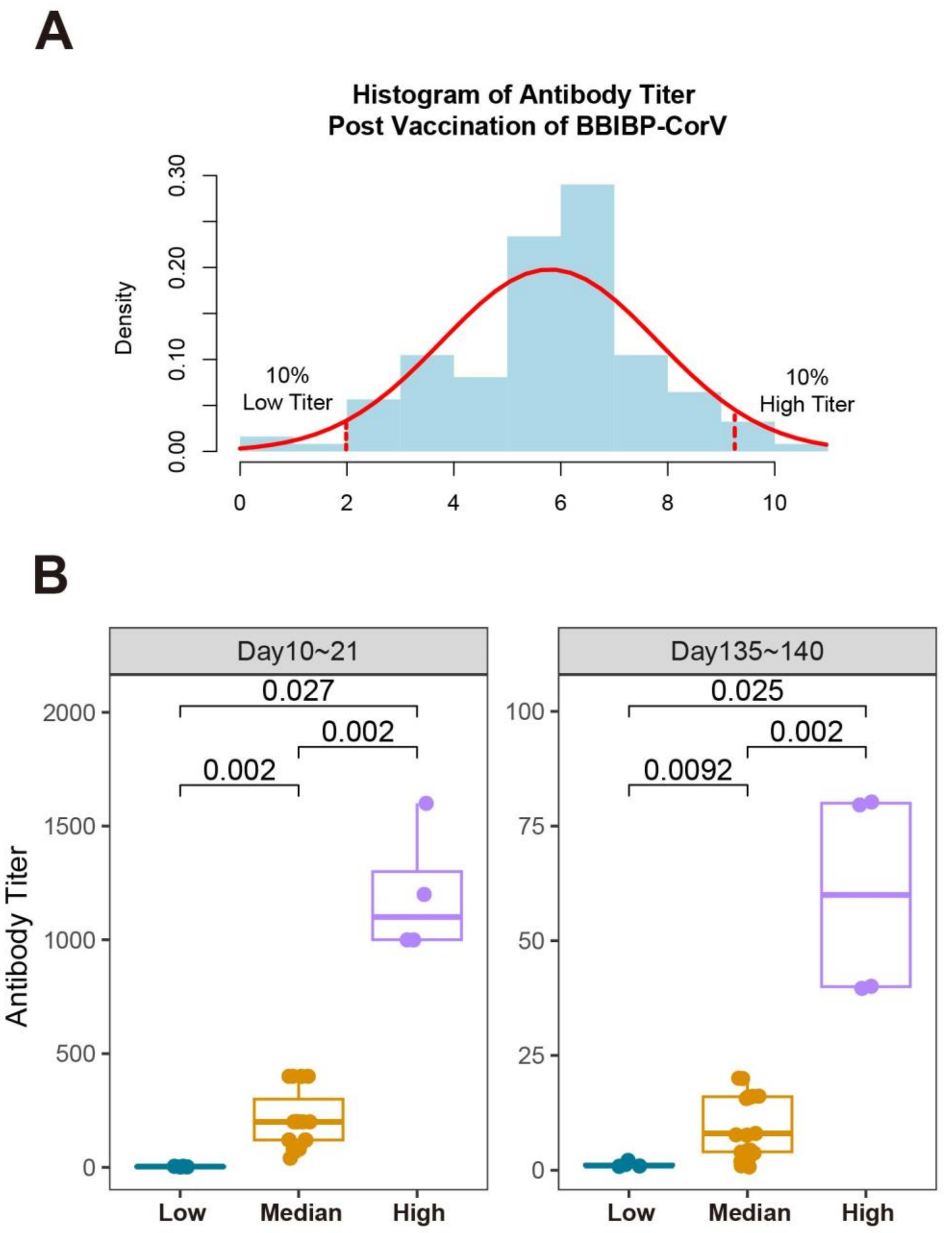

Figure S1. Antibody kinetics and titer distribution following BBIBP-CorV vaccination.(A) Distribution of IgG antibody titers during the decline phase (109-140 days post-vaccination) in the cohort (n=63). The density plot shows a normal distribution of antibody levels, with the lowest 10% (blue) and highest 10% (red) of individuals defined as low and high titer groups, respectively, and the intermediate 80% (gray) as the medium titer group.**(B)** Longitudinal comparison of antibody titers between early (10-21 days, left) and late (135-140 days, right) timepoints across titer groups. Box plots show median values with interquartile ranges, demonstrating the persistence of titer stratification over time. Statistical comparisons between groups are indicated above each plot (Low vs. Median: p=0.027; Low vs. High: p=0.002; Median vs. High: p=0.0092).

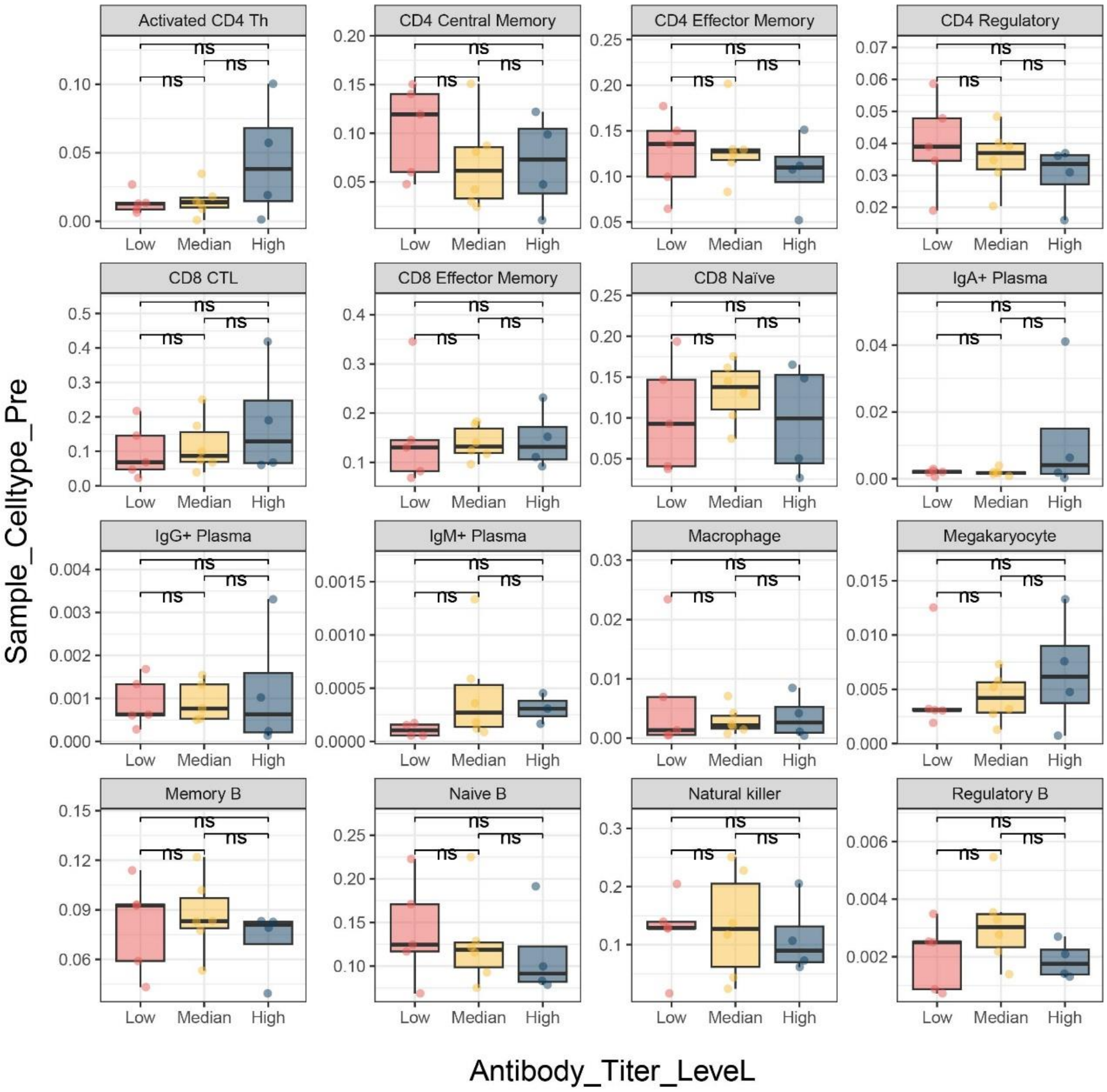

Figure S2: Distribution of immune cell type proportions across SARS-CoV-2 antibody titer levels. Boxplots show the proportion (Sample Celltype Precentage) of the rest 16 distinct immune cell types (as labeled) across samples grouped by low, median, and high antibody titer levels. Statistical significance was assessed using the Wilcoxon rank-sum test。

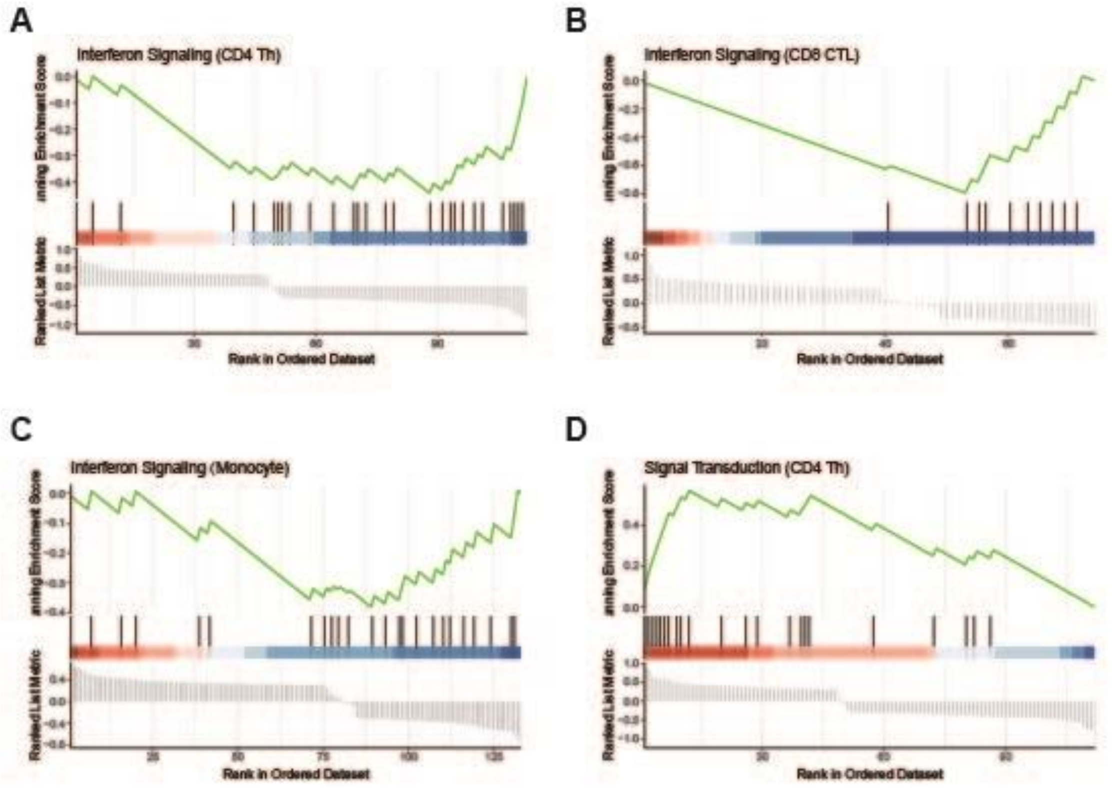

Figure S3. Gene Set Enrichment Analysis (GSEA) of Interferon Signaling and Signal Transduction Pathways in CD4+ T Helper Cells, CD8+ Cytotoxic T Lymphocytes, and Monocytes.

(A-C) GSEA plots depicting the enrichment of the interferon signaling pathway in three key immune cell types: $CD4^+$ T helper (Th) cells (A), $CD8^+$ cytotoxic T lymphocytes (CTLs) (B), and monocytes (C). Each plot illustrates the expression trend of this pathway as antibody titer increases. (D)A representative GSEA plot showing a positively enriched pathway (Signal Transduction) in $CD4^+$ Th cells, provided for comparison. Each panel is structured as follows: X-axis (Rank in Ordered Dataset): Represents the rank of all genes in the dataset, ordered from the most up-regulated (left) to the most down-regulated (right) in the high antibody titer group compared to the low titer group. Y-axis (Enrichment Score, ES): Represents the degree to which the genes of a predefined pathway (e.g., Interferon Signaling) are overrepresented at the top or bottom of the ranked list. Green Line (Enrichment Profile): Tracks the running enrichment score as the analysis moves down the ranked list of genes. A peak towards the left indicates enrichment of the pathway among up-regulated genes, while a peak towards the right indicates enrichment among down-regulated genes. Colored Barcode (Vertical Lines): Each vertical line in the barcode represents the position of a single gene from the pathway within the overall ranked

list. Red lines mark genes that are more highly expressed in the high titer group, while blue lines mark genes more highly expressed in the low titer group. Normalized Enrichment Score (NES): The NES is a normalized value that accounts for differences in gene set size and correlations in the dataset, allowing for comparison across different pathways. An NES > 0 indicates a trend of pathway upregulation, while an NES < 0 indicates a trend of pathway downregulation. The magnitude of the NES reflects the statistical strength of the enrichment.

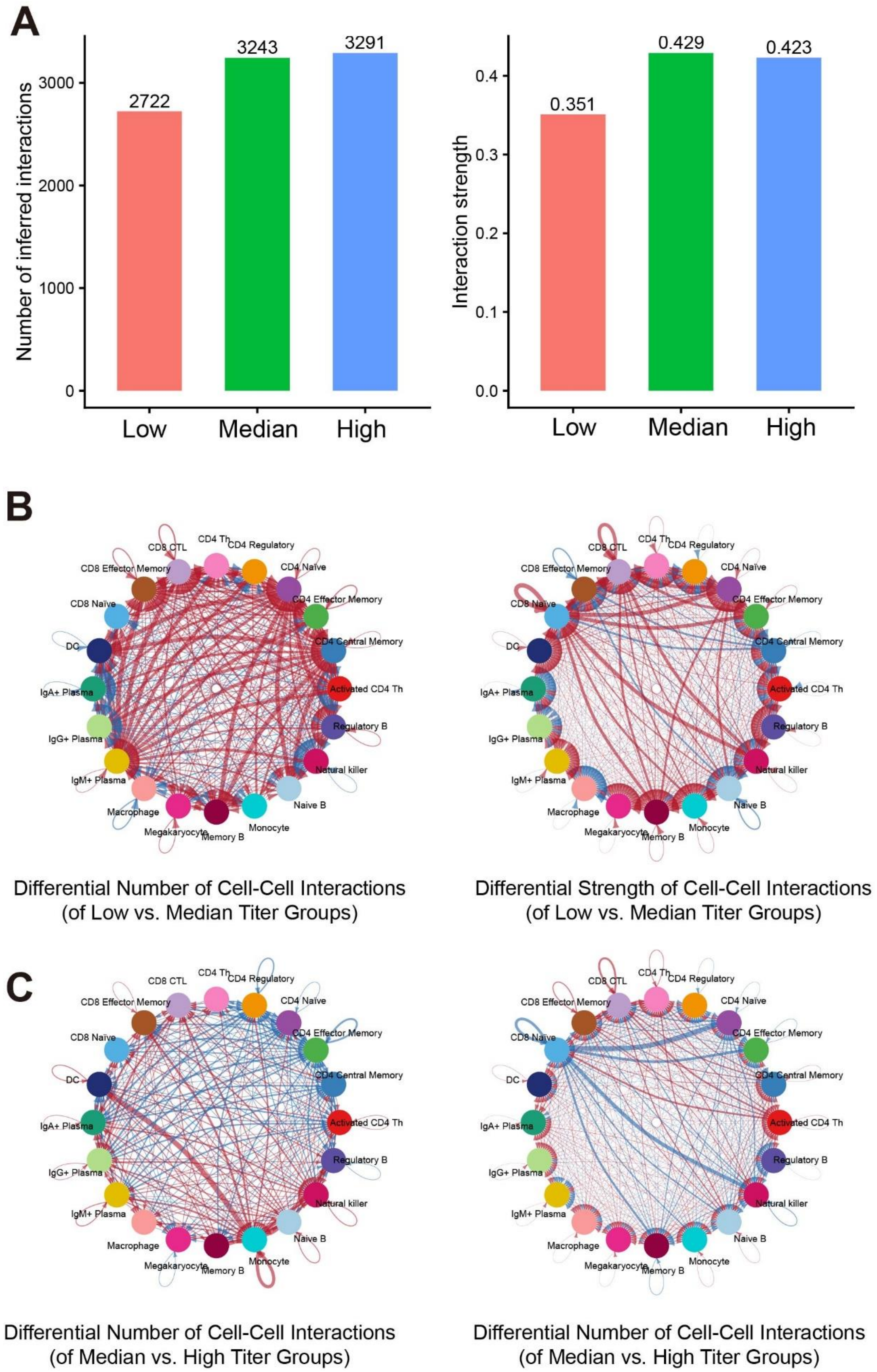

Figure S4: Cell-Cell Communication Networks Across SARS-CoV-2 Antibody Titer Groups.(A) Bar charts quantifying the total number (left) and overall strength

(right) of inferred cell-cell interactions within the low, median, and high titer groups. Numerical values represent the sum of all interactions per group. (B) Differential interaction analysis between the low and median titer groups. Circos plots depict the network of cell-cell communication, showing the difference in the number of interactions (left, measure="count") and interaction strength (right, measure="weight"). Nodes represent cell types (e.g., CD8 CTL, CD4 Naive). Red edges represent interactions that are increased in the median titer group relative to the low titer group, while blue edges represent decreased interactions. Edge width is proportional to the magnitude of the difference.(C) Differential interaction analysis between the median and high titer groups. The networks and edges are interpreted as in panel B, showing comparisons between the median and high titer groups. Cell-cell interactions were inferred and analyzed using the CellChat toolkit. Networks are based on the expression of ligand-receptor pairs.
